# Emotional Childhood Trauma Predicts Poorer Adherence in Inpatient Addiction Treatment

**DOI:** 10.1192/j.eurpsy.2026.10833

**Published:** 2026-07-31

**Authors:** J. Ristic-Ilic

**Affiliations:** Psychiatry, Railway Medical Centre Ljubljana, Ljubljana, Slovenia

## Abstract

**Introduction:**

Childhood traumatic experiences (CTEs) are highly prevalent among people with substance use disorders (SUDs). Most inpatient programmes screen for “any trauma,” but different trauma domains (emotional, physical, sexual, general) may have distinct implications for treatment retention. Clarifying which domains predict non-completion could make trauma-informed care more targeted and effective.

**Objectives:**

1. To test whether specific CTE domains differentially predict completion of inpatient SUD treatment.
2. To compare the predictive value of a binary “any-CTE” screen versus domain-level screening.
3. To explore whether risk-behaviour burden and admission anxiety/depression contribute to adherence.

**Methods:**

We conducted a cross-sectional study of consecutive inpatients at the Centre for the Treatment of Drug Addiction, University Psychiatric Clinic Ljubljana (13 January 2020–31 May 2021). During week 1, participants completed the Early Traumatic Inventory Self Report – Short Form, Alcohol Use Disorder Identification Test, and Zung Anxiety/Depression. Urine toxicology and hepatitis C virus serology were obtained; chart data captured comorbidities. The primary outcome was adherence, defined as completion of inpatient treatment according to the mutually agreed plan (yes/no). Associations between predictors and adherence were examined using multivariable logistic regression (p<0.05). Sample: N=217 (23.5% women; mean age 36±7 years).

**Results:**

CTEs were common (78.8%). A binary “any-CTE” indicator did not predict adherence. In domain-level analyses, emotional CTE independently predicted lower odds of completion (OR 0.52, 95% CI 0.30–0.90, p=0.019), whereas general, physical, and sexual CTEs were not significant. Reporting a protective adult at the time of trauma (30.4%) was not associated with adherence (OR 0.75, 95% CI 0.42–1.35, p=0.339). Although CTEs correlated with a higher count of risk behaviours (OR 1.35, 95% CI 1.17–1.57, p<0.001), risk-behaviour counts themselves did not predict adherence (OR 1.01, 95% CI 0.91–1.11, p=0.897). Admission anxiety and depressive symptoms showed no significant associations with adherence after adjustment.

**Conclusions:**

Retention risk in inpatient SUD care appears specifically linked to emotional childhood trauma rather than global trauma exposure, risk-behaviour burden, or baseline anxiety/depression. Routine domain-level trauma screening, flagging emotional CTE, could provide actionable “retention signals” to trigger early, individualized supports (e.g., enhanced alliance-building, stabilization, trauma-focused interventions). Prospective evaluations should test whether embedding such domain-level flags into admission workflows improves completion rates and post-discharge outcomes.

**Disclosure of Interest:**

None Declared

